# Study on distribution of sidewall earth pressure on open caissons considering soil arching effect

**DOI:** 10.1038/s41598-023-37865-9

**Published:** 2023-06-30

**Authors:** Bai Yang, Qingye Shi, Hexiang Zhou, Chao Qin, Weiwei Xiao

**Affiliations:** 1 School of Architecture and Transportation Engineering, Guilin University of Electronic Technology, Guilin, 541004 Guangxi China; 2 Faculty of Geosciences and Environmental Engineering, Southwest Jiaotong University, Chengdu, 610031 China; 3 Sichuan Road and Bridge Group Co., Ltd, Chengdu, 610041 Sichuan China

**Keywords:** Civil engineering, Structural geology

## Abstract

Based on the soil arching effect theory, the magnitude and distribution of sidewall earth pressure on open caissons when the embedded depth is large was analyzed by using theory of non-limit state earth pressure theory and horizontal differential element method. The theoretical formula was deduced. The theoretical calculation results are compared with the field test results and centrifugal model test results respectively. The results show that when the embedded depth of the open caisson is large, the distribution of earth pressure on the side wall of the open caisson first increases with the increase of embedded depth, reaches a peak value, and then sharply decreases. The peak point is located at 2/3 ~ 4/5 of the embedded depth. In engineering practice, when the embedded depth of the open caisson is 40 m, the relative error between the field test value and the theoretical calculation value is − 55.8% ~ 1.2%, with an average error of 13.8%. When the equivalent embedded depth of the open caisson in the centrifugal model test is 36 m, the relative error between the centrifugal model test value and the theoretical calculation value is − 20.1% ~ 68.0%, with an average error of 10.6%, The results are consistent well. The results of this article provides reference for the design and construction of open caisson.

## Introduction

Open caisson foundation has the characteristics of high bearing capacity, high stiffness, good integrity, etc., it is widely used in large-scale bridge engineering^1,2^. How to ensure the safe and steady process of open caisson sinking is a key issue in the construction of the open caisson foundation. In order to solve the above problem, it is necessary to conduct in-depth research on the magnitude and distribution of the sidewall earth pressure of the open caisson.

At present, the design of open caisson is mainly calculated by standard^3^, and the distribution law of sidewall friction is mainly based on the analysis results of sinking mechanism of large diameter pile and small open caisson^4^. The existing field monitoring and laboratory test results^5–13^ all indicate that the above calculation method is not applicable to large open caisson foundations. In the field test, Chen XP^5^ conducted real-time monitoring of the whole sinking process of the open caisson on a certain bridge's main pier, and preliminary analyzed the sinking mechanism and mechanical characteristics of the open caisson, and the magnitude and distribution of sidewall earth pressure at different depths of the open caisson are obtained. On the basis of the magnitude and distribution law, a calculation model for sinking resistance of the open caisson is established. In order to obtain the variation process of soil pressure during the first sinking stage of a large caisson, Jiang F et al.^6^ used finite element method to simulate the caisson foundation of the Changtai Yangtze River Bridge. Combined with field measured data of soil pressure at the blade foot, the variation law of soil pressure at the blade foot during the soil sampling process was obtained. Guo MW et al.^7^ proposed a new method for calculating the resistance at the end of the caisson based on the blade foot soil pressure. Chen BG et al.^8^ obtained the expression equation of caisson movement process by establishing the caisson settlement model, and revealed the kinematics characteristics and influence mechanism of caisson settlement. Lv CJ et al.^9^ conducted real-time monitoring of the sinking process of large open caissons in clay sand interactive formations, and further studied the magnitude and distribution of the reaction force, sidewall pressure, and lateral friction during the sinking process. In terms of indoor model tests, Wang Jian^11^ and Mu Bao-gang^13^ successively carried out model tests on sinking resistance of the open caisson, and studied the change characteristics of sidewall friction resistance, subsidence range of soil surface outside the wall, flow trend of soil particles and so on when the open caisson sank to different depths. At present, less research results in theoretical terms.

The soil arching effect is the phenomenon that the stress in the soil is transferred from the yield region to the adjacent unyield region^14^. In 1943, Terzaghi^15^ proved the existence of soil arching effect for the first time through a trapdoor test. Later, in the study of earth pressure on earth retaining walls, Handy^16^, Zhao XY^17^, Liu H^18^, Lai F^19^ and Zhang H^20^, etc. considered the soil arching effect and obtained the calculation method of earth pressure. Jiang XD^21^ applied the theory of soil arching effect to the sinking calculation of the open caisson, and derived the coefficient of sidewall earth pressure and the calculation formula of sidewall earth pressure. However, it did not take into account the stress relaxation effect of the soil outside the cutting curb^5^, and there was no theoretical basis for the assumption that the active and passive limit states are above and below the critical depth, respectively.

The non-limiting state earth pressure theory is improved from the Rankine or Coulomb earth pressure theory, which is widely used in the calculation of earth pressure on earth retaining wall^22,23^. The theory believes that the soil is a gradual process from the static state to ultimate state, and in the calculation of earth pressure, the influence of the displacement mode of retaining wall and displacement on the magnitude and distribution of earth pressure is considered. Similarly, the posture change during sinking process of the open caisson can also be regarded as a displacement of the sidewall relative to the soil.

In this paper, based on the soil arching effect theory, the magnitude and distribution of sidewall earth pressure on open caissons when the depth of sinking is large was analyzed by using theory of non-limit state earth pressure theory and horizontal differential element method. The theoretical formula was deduced. The theoretical calculation results are compared with the field test results and centrifugal model test results respectively. The results can provide references for the design and construction of open caissons.

## Considering the internal and external friction angle of sidewall displacement

Taking the open caisson sinking in soil with a horizontal non-cohesive soil surface as an example, in order to simplify the calculation, the working condition of the open caisson in a vertical state is taken for analysis, that is, the sidewall transversals relative to the soil.

In the non-limit state, there is a quasi-slip surface in the soil. It is assumed that the quasi-slip surface is straight and the sidewall moves away from the soil, the angle between the quasi-slip surface and the sidewall is $\beta = \frac{\pi }{4} - \frac{\varphi }{2}$, and when the sidewall transversals towards the soil, the angle between the quasi-slip surface and the wall is $\beta = \frac{\pi }{4} + \frac{\varphi }{2}$. At this time, the internal friction angle $\varphi_{m}$ of soil is between the initial internal friction angle $\varphi_{0}$ and the ultimate internal friction angle $\varphi$, which can be calculated by Eq. (1)^24^

1 $$ \varphi_{{\text{m}}} = \arctan \left[ {\tan \varphi_{0} + K_{{\text{d}}} \,\left( {\tan \varphi - \tan \varphi_{0} } \right)} \right]. $$

Type: $K_{{\text{d}}} = 4\arctan (S/S_{{\text{c}}} )/\pi$; $S$ is the actual translational displacement of the sidewall; $S_{{\text{c}}}$ is the critical displacement of sidewall when the soil reaches the active or passive limit state.

When the sidewall transversals horizontally, the corresponding displacement when the sand reaches the active limit state is about 0.001*H* ~ 0.005*H*, and when it reaches the passive limit state is about 0.05*H*. (*H* is the buried depth of open caisson).

Considering the influence of friction angle $\delta$ at the interface between soil and sidewall, $\varphi_{0}$ can be solved by Eq. (2)^23^.

2 $$ \frac{1}{{K_{0} }} = \left[ {\frac{1}{{\cos \varphi_{0} }} + \left( {\tan^{2} \varphi_{0} + \tan \varphi_{0} \tan \delta } \right)^{\frac{1}{2}} } \right]^{2} $$

For normally consolidated soil, $K_{0} = 1 - \sin \varphi$ and to be conservative, to take $\delta { = }\varphi /2$.

## Soil arching effect outside the sidewall of open caisson

### Stress model of principal stress arch

**Figure 1:**
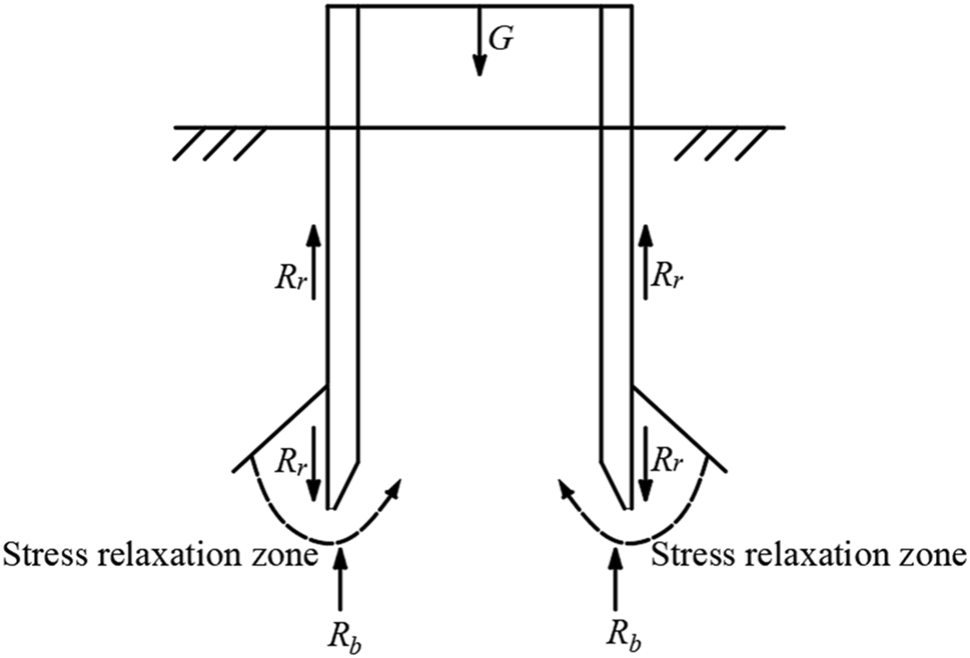
Schematic diagram of stress relaxation effect.

When the open caisson sinks to a certain depth, due to excavation, the height of the soil surface inside the well is lower than that outside the well, and a soil pressure difference is formed inside and outside the cutting curb, which makes the soil outside the cutting curb have a tendency to migrate to the inside and form a pressure relaxation area in the soil, as shown in Fig. 1.Therefore, the soil outside the sidewall can be divided into two parts, non-stress relaxation zone and stress relaxation zone, from top to bottom.

**Figure 2:**
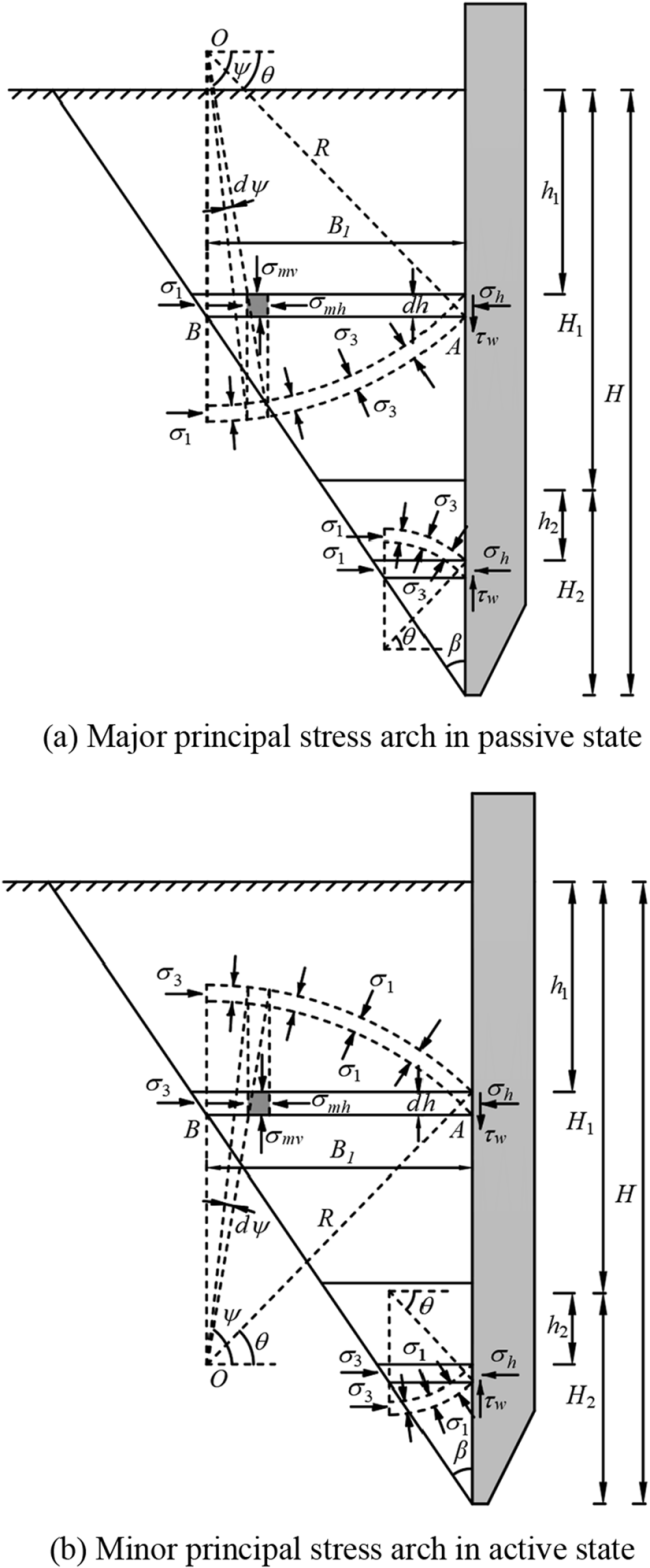
Soil arching effect outside of sidewall.

When the sinking depth of the open caisson is large, the sinking rate of the open caisson sucking sludge is small or in a stagnant state. At this time, the soil in the non-stress relaxation zone moves upward relative to the sidewall and is affected by the downward friction of the sidewall, while the soil mass in the stress relaxation zone migrates to the inside of the open caisson through the cutting curb under the action of sucking sludge in the well, and thus is affected by the upward friction of the sidewall. According to the principle of soil arching effect, under the action of the sidewall friction, the soil stress outside the sidewall deflected, causing the stress in the yielding area to transfer to the adjacent unyielding area, forming the principal stress arch between the quasi-slip fracture surface and the sidewall. If the sidewall moves away from the soil, the soil is in an active state, forming a minor principal stress arch. If the sidewall moves toward the soil, the soil is in a passive state, forming a major principal stress arch. The calculation model is shown in Fig. 2. The sinking depth of the open caisson is *H*, the height of the non-stress relaxation area is $H_{1}$, and the height of the stress relaxation area is $H_{2}$.

### Stress analysis of principal stress arch

The differential element body at point A establishes the horizontal and vertical balance equations. In the passive state:

3 $$ \sigma_{h} = \sigma_{1} \sin^{2} \theta + \sigma_{3} \cos^{2} \theta $$

4 $$ \tau_{w} = \,\left( {\sigma_{1} - \sigma_{3} } \right)\sin \theta \cos \theta $$

5 $$ K_{{\text{p}}} = \sigma_{1} /\sigma_{3} $$

In the active state:

6 $$ \sigma_{h} = \sigma_{1} \cos^{2} \theta + \sigma_{3} \sin^{2} \theta $$

7 $$ \tau_{w} = \,\left( {\sigma_{1} - \sigma_{3} } \right)\sin \theta \cos \theta $$

8 $$ K_{{\text{a}}} = \sigma_{3} /\sigma_{1} $$

where $\theta$ is the included angle between $\sigma_{3}$ and the horizontal direction; $\sigma_{h}$ is the horizontal stress; $\tau_{w}$ is the shear stress; $K_{{\text{p}}}$ is the Rankine passive earth pressure coefficient; $K_{{\text{a}}}$ is the Rankine active earth pressure coefficient.

**Figure 3:**
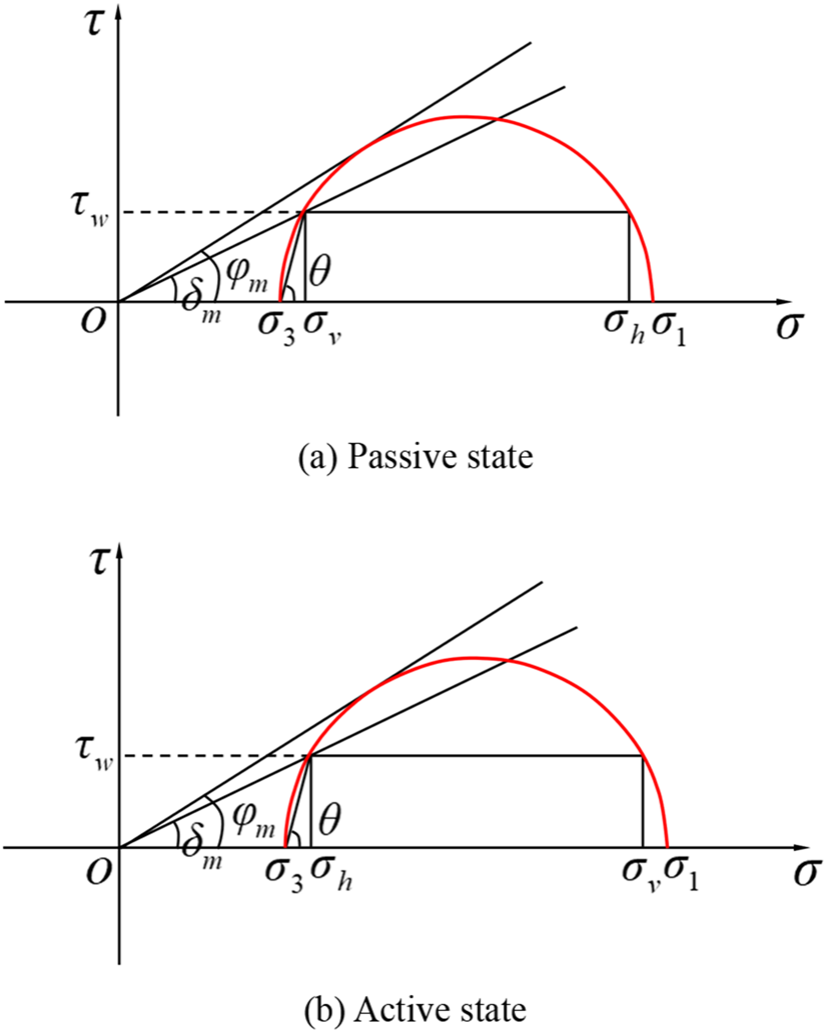
Mohr stress circle of the soil outside of sidewall.

As can be seen from the Mohr stress circle in Fig. 3, whether in the passive state or the active state, there is:

9 $$ \sigma_{h} = \sigma_{1} + \sigma_{3} - \sigma_{v} $$

Type: $\sigma_{v}$ is the vertical stress.

The passive states of simultaneous (3), (5) and (9) can be obtained:

10 $$ \frac{{\sigma_{{\text{h}}} }}{{\sigma_{3} }} = K_{{\text{p}}} \sin^{2} \theta + \cos^{2} \theta $$

11 $$ \frac{{\sigma_{{\text{v}}} }}{{\sigma_{3} }} = K_{{\text{p}}} \cos^{2} \theta + \sin^{2} \theta $$

The active states of the simultaneous (6), (8) and (9) can be obtained:

12 $$ \frac{{\sigma_{{\text{h}}} }}{{\sigma_{1} }} = K_{{\text{a}}} \sin^{2} \theta + \cos^{2} \theta $$

13 $$ \frac{{\sigma_{{\text{v}}} }}{{\sigma_{1} }} = K_{{\text{a}}} \cos^{2} \theta + \sin^{2} \theta $$

Dividing Eq. (10) by Eq. (11) and Eq. (12) by Eq. (13), can be obtained, whether active or passive state, the theoretical lateral pressure coefficient is:

14 $$ K = \frac{{\sigma_{{\text{h}}} }}{{\sigma_{{\text{v}}} }} = \frac{{K\sin^{2} \theta + \cos^{2} \theta }}{{K\cos^{2} \theta + \sin^{2} \theta }} $$

Type: $K$ is the earth pressure coefficient, $K_{{\text{p}}}$ is the passive earth pressure coefficient in the passive state, and $K_{{\text{a}}}$ is the active earth pressure coefficient in the active state.

The friction angle $\delta$ of the interface between the soil and the sidewall is known, and it can be derived from Fig. 3 that the value of $\theta$ in the passive state is:

15 $$ \tan \delta = \frac{{\left( {K_{{\text{p}}} - 1} \right)\tan \theta }}{{K_{{\text{p}}} + \tan^{2} \theta }} $$

Similarly, the value of $\theta$ in the active state is:

16 $$ \tan \delta = \frac{{\left( {1 - K_{{\text{a}}} } \right)\tan \theta }}{{1 + K_{{\text{a}}} \tan^{2} \theta }} $$

### Shape of stress arch

Although the theoretical soil arch curve has been proved to be a part of catenary^25^, its expression is more complicated and can generally be simplified as a circular arc soil arch for calculation^26,27^.

Taking the non-stress relaxation area as an example, assuming that the stress arch trace is a circular arc, as shown in Fig. 2, point A is the coordinate origin, the x-axis is positive horizontally to the outside of the wellbore, and the y-axis is positive vertically. The relative coordinates of the center of the circular arc arch are ($B_{1}$,$B_{1} \tan \theta$), then the geometric equation of the circular arc arch is:

17 $$ \left( {x - B_{1} } \right)^{2} + \,\left( {y - B_{1} \tan \theta } \right)^{2} = \frac{{B_{1}^{2} }}{{\cos^{2} \theta }} $$

Type: $B_{1}$ is the horizontal distance between the center of the circular arc arch and the sidewall.

### Practical lateral earth pressure coefficient

As shown in Fig. 2, it can be known from the geometric relationship:

18 $$ \frac{\cos \psi }{{B_{1} - x}} = \frac{\cos \theta }{{B_{1} }} $$

Type: $\psi$ is the included angle between the center line of the circular arc arch differential element and the horizontal direction.

It can be obtained from the Molar stress circle, in the passive state:

19 $$ \frac{{\sigma_{{{\text{mh}}}} }}{{\sigma_{{3}} }} = K_{{\text{p}}} \sin^{2} \psi + \cos^{2} \psi $$

20 $$ \frac{{\sigma_{{{\text{mv}}}} }}{{\sigma_{{3}} }} = K_{{\text{p}}} \cos^{2} \psi + \sin^{2} \psi $$

Type: $\sigma_{mh}$ is the horizontal stress on the circular arc arch differential element; $\sigma_{mv}$ is the vertical stress on the circular arc arch differential element.

Dividing Eq. (19) by Eq. (20), the practical lateral pressure coefficient in the passive state can be known as:

21 $$ K_{{{\text{pw}}}} = \frac{{\sigma_{{\text{h}}} }}{{\overline{\sigma }_{{{\text{v1}}}} }} = \frac{{{{\sigma_{{\text{h}}} } \mathord{\left/ {\vphantom {{\sigma_{{\text{h}}} } {\sigma_{{3}} }}} \right. \kern-0pt} {\sigma_{{3}} }}}}{{{{\overline{\sigma }_{{{\text{v1}}}} } \mathord{\left/ {\vphantom {{\overline{\sigma }_{{{\text{v1}}}} } {\sigma_{{3}} }}} \right. \kern-0pt} {\sigma_{{3}} }}}} = \frac{1}{{{{\overline{\sigma }_{{{\text{v1}}}} } \mathord{\left/ {\vphantom {{\overline{\sigma }_{{{\text{v1}}}} } {\sigma_{{3}} }}} \right. \kern-0pt} {\sigma_{{3}} }}}}\left( {K_{{\text{p}}} \sin^{2} \theta + \cos^{2} \theta } \right) $$

Type: $\overline{\sigma }_{v1}$ is the average vertical stress on the horizontal differential element, which can be calculated by $\overline{\sigma }_{{{\text{v1}}}} = \frac{{\int_{0}^{{B_{{1}} }} {\sigma_{{{\text{mv}}}} dx} }}{{B_{{1}} }}$.

Simultaneous Eqs. (18) and (20) and calculating through integrals can be obtained:

22 $$ \frac{{\overline{\sigma }_{{{\text{v1}}}} }}{{\sigma_{{3}} }} = \frac{{\left( {K_{{\text{p}}} - 1} \right)\cos^{2} \theta_{{1}} }}{3} + 1 $$

Substitute into Eq. (21) to get:

23 $$ K_{{{\text{pw}}}} = \frac{1}{{\frac{{\left( {K_{{\text{p}}} - 1} \right)\cos^{2} \theta }}{3}{ + }1}}\,\left( {K_{{\text{p}}} \sin^{2} \theta + \cos^{2} \theta } \right) $$

Similarly, the practical lateral pressure coefficient in the active state is:

24 $$ K_{{{\text{aw}}}} = \frac{1}{{\frac{{\left( {K_{{\text{a}}} - 1} \right)\cos^{2} \theta }}{3}{ + }1}}\,\left( {K_{{\text{a}}} \sin^{2} \theta + \cos^{2} \theta } \right) $$

Comparing Eq. (23) and Eq. (24), it can be seen that regardless of the passive state or the active state, the practical lateral pressure coefficient is:

25 $$ K_{{\text{w}}} = \frac{1}{{\frac{{\left( {K - 1} \right)\cos^{2} \theta }}{3}{ + }1}}\left( {K\sin^{2} \theta + \cos^{2} \theta } \right) $$

## Earth pressure based on horizontal differential element method

### Establishment of basic equations

#### Non-stress relaxation area

**Figure 4:**
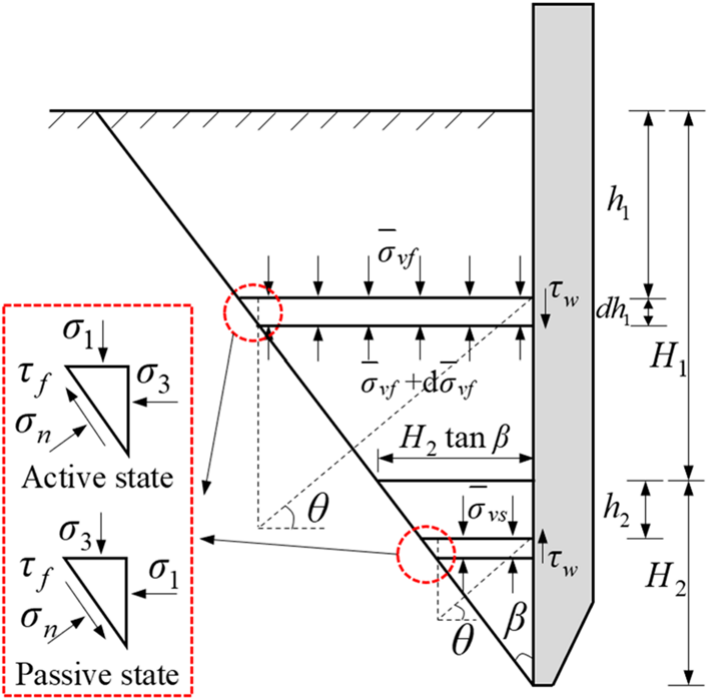
Stress of horizontal differential element outside of sidewall.

Take a horizontal differential element with thickness $dh$ at the distance $h_{1}$ from the surface of the non-stress relaxation area, and the force acting on the horizontal differential element is shown in Fig. 4.

From the equilibrium conditions of the horizontal differential element in the vertical direction, it can be obtained:

26 $$ \left( {H_{{1}} { + }H_{{2}} - h_{{1}} } \right)\tan \beta \left( {\frac{{d\overline{\sigma }_{{{\text{vf}}}} }}{{dh_{{1}} }}} \right) - \overline{\sigma }_{{{\text{vf}}}} K_{{\text{w}}} \tan \delta = \gamma \,\left( {H_{{1}} { + }H_{{2}} - h_{{1}} } \right)\tan \beta $$

Type: $\gamma$ is the soil weight; $\overline{\sigma }_{vf}$ is the average vertical stress on the horizontal differential element in the non-stress relaxation area; $\beta$ is the included angle between the quasi-slip surface and the sidewall.

Taking the boundary condition as $\overline{\sigma }_{{{\text{vf}}}} (h_{{1}} = 0) = 0$ , the special solution of $\overline{\sigma }_{vf}$ is obtained by Eq. (26):

27 $$ \overline{\sigma }_{{{\text{vf}}}} = \frac{{\gamma \left( {A - h_{{1}} \cot \beta } \right)}}{B\cot \beta }\,\left[ {\left( {\frac{A}{{A - h_{{1}} \cot \beta }}} \right)^{B} - 1} \right] $$

Type: $$A = (H_{{2}} + H_{{1}} )\cot \beta$$; $$B = 1 + K_{{\text{w}}} \tan \delta \cot \beta$$.

Earth pressure on the sidewall of the non-stress relaxation area:

28 $$ p_{{1}} = \frac{{K_{{\text{w}}} \gamma \left( {A - h_{{1}} \cot \beta } \right)}}{B\cot \beta }\,\left[ {\left( {\frac{A}{{A - h_{{1}} \cot \beta }}} \right)^{B} - 1} \right] $$

#### Stress relaxation area

Similarly, the vertical equilibrium equation of the force on the horizontal differential element at the distance $h_{2}$ from the top surface of the stress relaxation area is as follows:

29 $$ (H_{{2}} - h_{{2}} )\tan \beta \left( {\frac{{d\overline{\sigma }_{{{\text{vs}}}} }}{{dh_{{2}} }}} \right) + \overline{\sigma }_{{{\text{vs}}}} K_{{\text{w}}} \tan \delta = \gamma \left( {H_{{2}} - h_{{2}} } \right){\text{tan}}\beta $$

Type: $\overline{\sigma }_{vs}$ is the average vertical stress on the horizontal differential element in the stress relaxation area.

Taking the boundary condition as $\overline{\sigma }_{{{\text{vf}}}} (h_{{1}} = H_{{1}} ) = \overline{\sigma }_{{{\text{vs}}}} (h_{{2}} = 0)$, and the special solution of $\overline{\sigma }_{vs}$ is obtained from Eq. (22):

30 $$ \overline{\sigma }_{{{\text{vs}}}} { = } - \frac{{\gamma (H_{{2}} - h_{{2}} )}}{C} + D(\frac{{H_{{2}} - h_{{2}} }}{{H_{{2}} }})^{1 - C} $$

Type: $$C = 1 - K_{{\text{w}}} \tan \delta \cot \beta$$; $$D = \overline{\sigma }_{{{\text{vf}}}} \,\left( {h_{{1}} = H_{{1}} } \right) + \frac{{\gamma H_{{2}} }}{C}$$.

Earth pressure on the sidewall of the stress relaxation area:

31 $$ p_{{2}} { = } - K_{{\text{w}}} \left[ {\frac{{\gamma \left( {H_{{2}} - h_{{2}} } \right)}}{C} - D\,\left( {\frac{{H_{{2}} - h_{{2}} }}{{H_{{2}} }}} \right)^{1 - C} } \right] $$

### Range of stress relaxation area

The height of the open caisson stress relaxation area can be determined by referring to existing field monitoring data. The research results of Chen XP^5^ showed that the range of stress relaxation area is 1-10 m, and the upper limit is taken when the sinking depth is large.

## Examples verification

### On-site monitoring of open caisson sinking of main pier of a bridge

The size of the open caisson of the main pier of a bridge is 86.9 m × 58.7 m, the radius of the inverted circle is 7.45 m, and the height is 115 m.The open caisson is built in water and needs to pass through dense silty sand layer, dense fine sand layer and dense coarse sand layer successively when sinking. According to the field geological survey data, the saturation weight density of soil is $\gamma = 19.2{\text{kN/m}}^{{3}}$, and the angle of internal friction is $\varphi = 36.9^\circ$. To be conservative, the angle of friction between the soil and the sidewall is $\delta = 18.5^\circ$.

**Figure 5:**
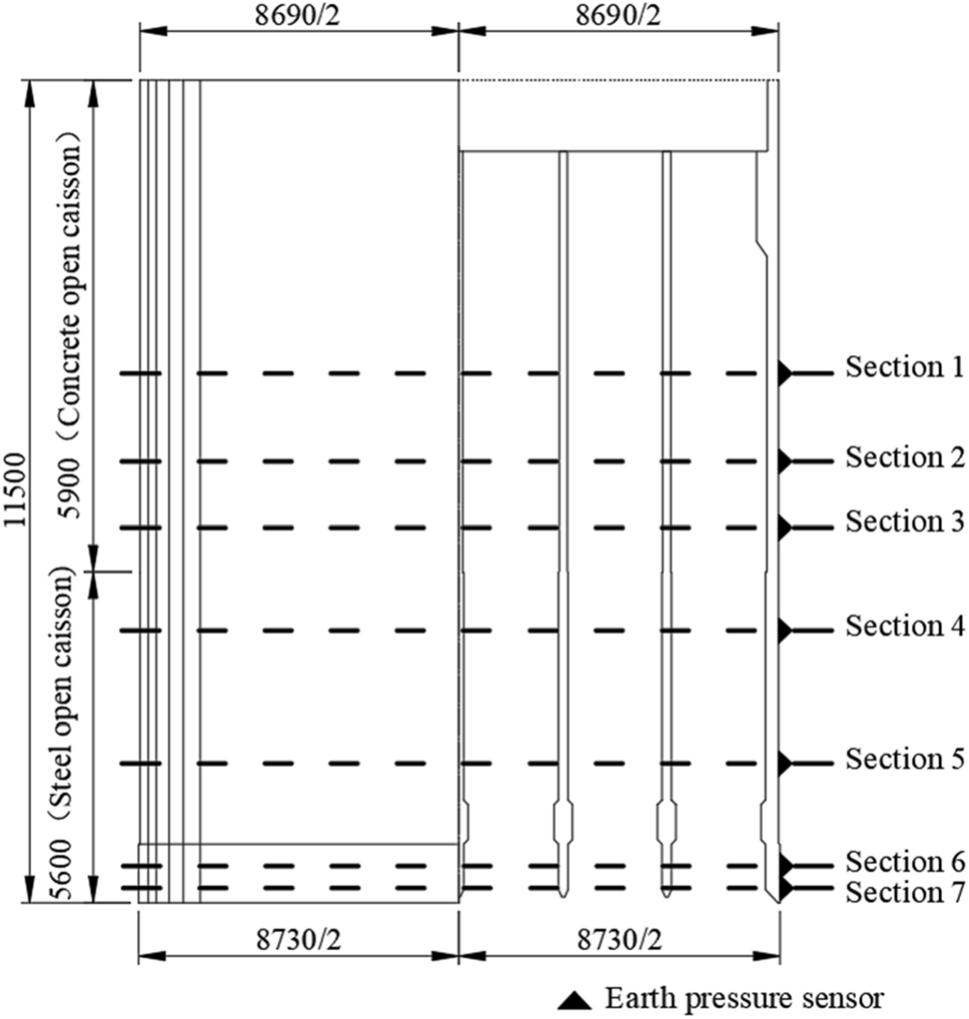
Monitoring sections of earth pressure sensor in sidewall (unit: cm).

**Figure 6:**
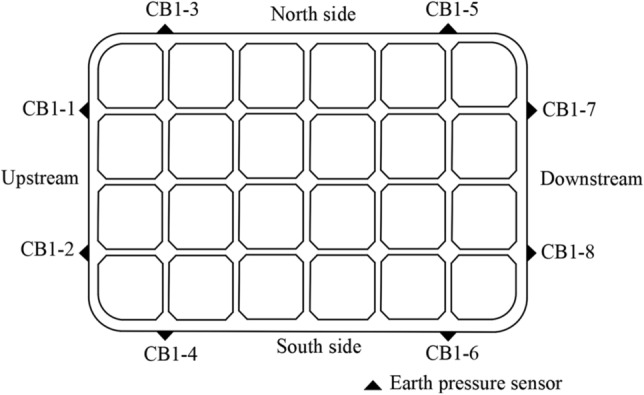
Plane layout diagram of earth pressure sensor in sidewall.

Seven monitoring sections were set at different heights around the outer wall of the open caisson. The sections were 2 m, 5 m, 19 m, 37 m, 51 m, 60 m and 72 m above the cutting curb, and is divided into section 1–section 7 according to the elevation of the section from high to low. The section layout of the sidewall earth pressure sensor is shown in Fig. 5, and the plane layout of each section is shown in Fig. 6.

Due to the limitation construction conditions of the open caisson was built in the water and traversed a huge thick soil layer, many earth pressure sensors were damaged and failed after entering the soil, and only some of the sensors in Sections “Earth pressure based on horizontal differential element method” and “Conclusions and suggestions” were intact. Selecting the working condition of the open caisson embedded depth *H*≈100 m and the open caisson is vertical to the north–south direction for analysis. At this time, the stress relaxation area $h_{2}$ of the open caisson is 10 m reaches the passive limit state and the displacement $S_{{\text{c}}}$ is 200 cm.

**Figure 7:**
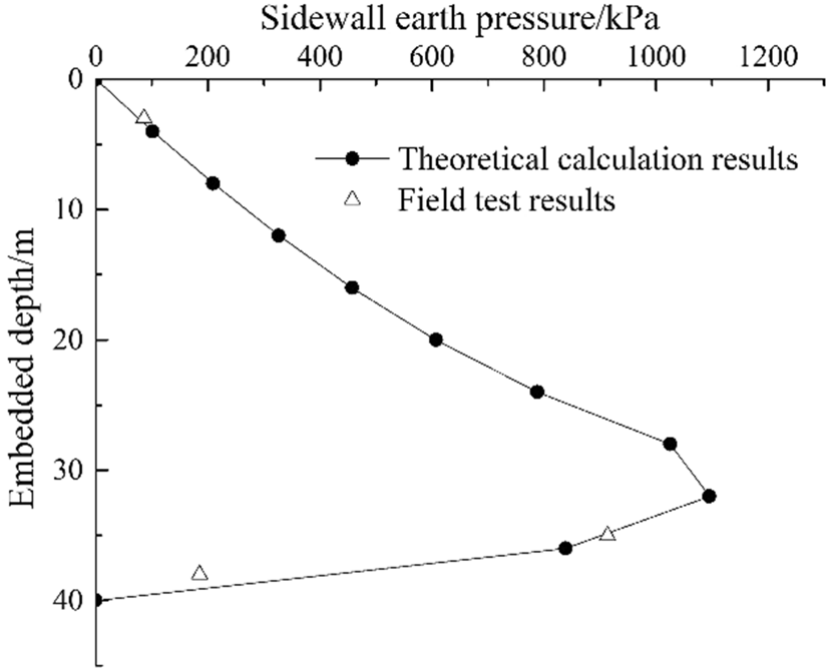
Comparison between the calculated results of this paper and the field measured results.

According to the field measurement, the cumulative translational displacement in the south direction of the open caisson is $S = 72.3{\text{cm}}$, the soil on the south side of the open caisson is in a passive state. The calculation results are compared with the field test results, as shown in Fig. 7. The calculated results of the method in this paper is basically consistent with the field measured results. There are few field test results. From the theoretical calculation curve, it can be seen that the sidewall earth pressure first increases with the increase of embedded depth, reaches a peak value, and then sharply decreases. The peak point is 32 m, which is 80.0% of the embedded depth.

The theoretical method was used to calculate the earth pressure of Sections “Earth pressure based on horizontal differential element method” and “Conclusions and suggestions” of the caisson. The calculated values were compared with the field values, and the results are shown in Table 1. The relative error between the field value and the theoretical calculation value is -55.8% ~ 1.2%, with an average error of 13.8%.

**Table 1:** Comparison between theoretical calculation results and field test results.

Embedded depth/m	Theoretical calculation results/kPa	Field test results/kPa	Relative error/%
3.0	75.9	85.8	13.1
35.0	902.7	913.8	1.2
38.0	419.2	185.2	-55.8

### Centrifugal model test of sinking resistance of open caisson

**Figure 8:**
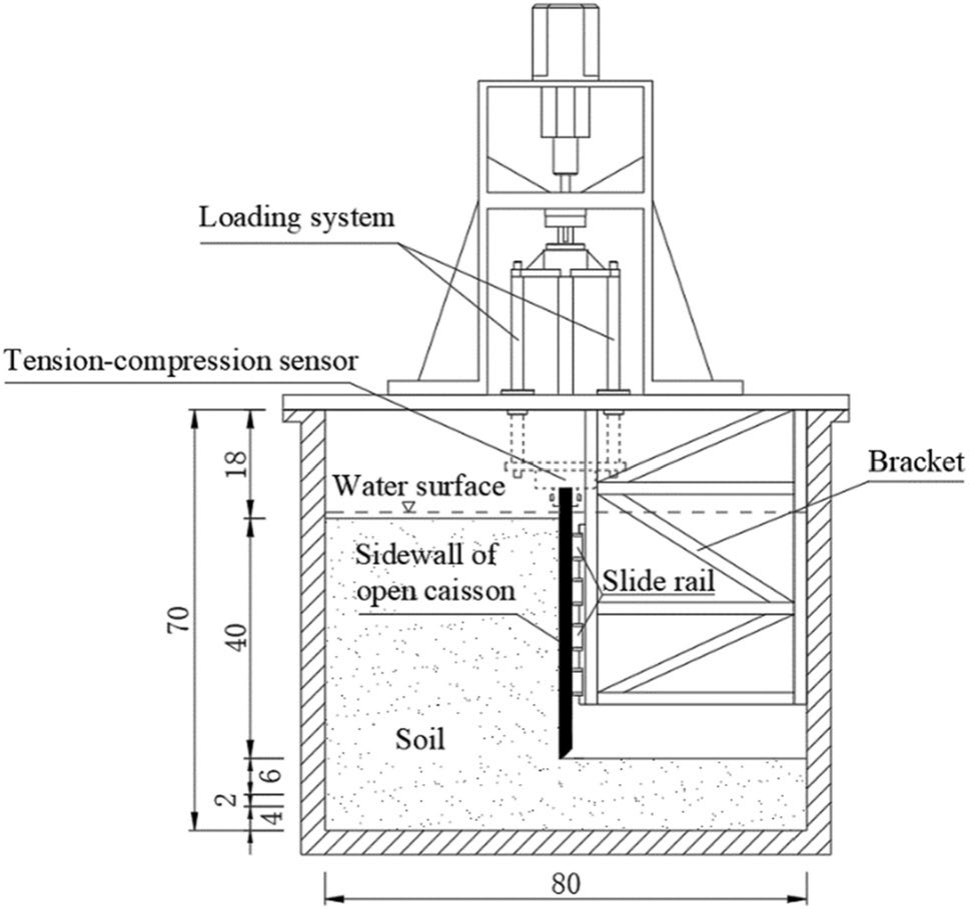
Layout diagram of centrifuge model test (unit: cm).

The centrifugal model test of the open caisson sinking is used to study the distribution law of earth pressure on the sidewall of open caisson, the outer wall of open caisson is selected as the research object, and the three-dimensional model of the open caisson is simplified into a two-dimensional model of the sidewall. The sidewall model of 45 cm high, 70 cm wide, the width of the cutting curb tread surface is 0.3 cm, the width of the cutting curb slope is 1.7 cm, and the inclination angle is 45°. The open caisson is connected with a fixed bracket by a slide rail and can slide freely in the vertical direction after installation, as shown in Fig. 8.

It is very difficult to dynamically simulate the process of mud suction and sinking of open caisson under the current technical conditions of the centrifugal model test technology. In the test, the working condition of the open caisson embedded depth *H*≈36 m was selected, and the dynamic analysis was replaced by the quasi-static analysis. In addition, the loading system is used to control the vertical force to be constant to simulate the process that the vertical force balance of the open caisson is broken after a certain mud suction, and the open caisson is sank and reached rebalance by the action of self-weight stress. The centrifugal acceleration was set to 90 g, and the initial position of the cutting curb tread surface is level with the soil surface in the well.

Silt sand was selected as the test soil, the saturation weight density of soil $\gamma = 19.8{\text{kN/m}}^{{3}}$, and internal friction angle $\varphi = 36.2^\circ$. For the sake of conservativeness, take $\delta = 18.1^\circ$.

**Figure 9:**
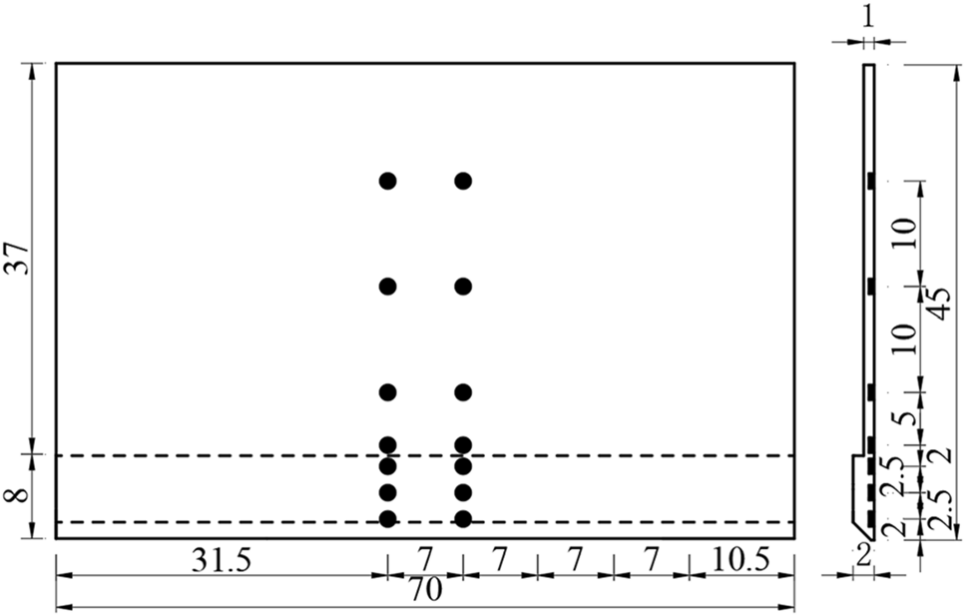
Layout diagram of earth pressure sensor in sidewall (unit: cm).

The earth pressure sensor is used to measure the earth pressure on the sidewall of the open caisson. The arrangement of the earth pressure sensor is shown in Fig. 9.

**Figure 10:**
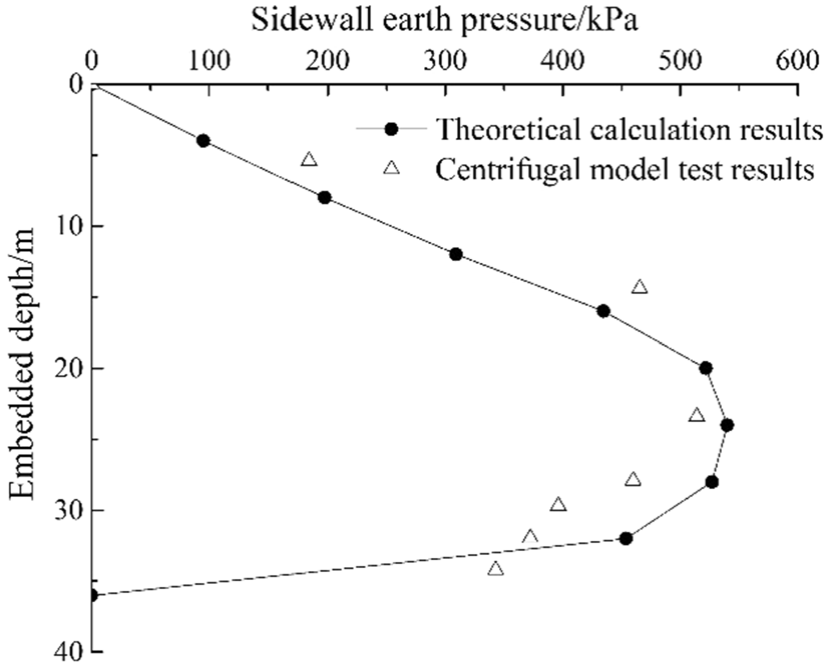
Comparison between calculated results of this paper and centrifuge model test results.

Due to the limited machining accuracy, when riveting the open caisson model with the loading system, it is necessary to make a slight adjustment of fine-tune the surface of the open caisson model towards the direction of soil, the adjustment value is about 0.5 cm. After the conversion by similarity ratio, $S = 45{\text{cm}}$, when the displacement reaches the passive limit state, $S_{{\text{c}}} = 180{\text{cm}}$.The soil is in a passive state. Assuming that the stress relaxation area of the open caisson is 18 m, the calculated results of the method in this paper is compared with the results of centrifugal model test, the results are shown in Fig. 10. The calculation results of the method in this paper are in good agreement with the results of the centrifugal model test. The stress relaxation area of the centrifugal model is greater than 10 m, the reason is that when the test data are collected, the open caisson has not appeared obvious subsidence, resulting in a larger range of the soil with downward displacement relative to the sidewall, that is, a larger range of stress relaxation area. The equivalent embedded depth of the open caisson in the centrifugal model test is 36 m. From the theoretical calculation results curve and centrifugal model test results, it can be seen that the earth pressure shows a distribution characteristic of first increasing with the increase of embedded depth, reaching a peak value and then sharply decreasing. The theoretical calculation peak point is 24 m, and the centrifugal model test peak point is 23.4 m, which are 66.7% and 65.0% of the embedded depth, respectively.

The theoretical method was used to calculate the earth pressure of the sensor burial depth in the centrifugal model test. The calculated value was compared with the experimental value, and the results are shown in Table 2. The error between the experimental value and the theoretical calculation value is − 20.1% ~ 68.0%, with an average error of 10.6%.

**Table 2:** Comparison between theoretical calculation results and centrifugal model test results.

Embedded depth/m	Theoretical calculation results/kPa	Centrifugal model test results/kPa	Relative error/%
5.4	131.1	184.0	40.4
14.4	384.4	465.5	21.1
23.4	537.4	514.2	− 4.3
27.9	527.5	460.0	− 12.8
29.7	495.9	396.1	− 20.1
31.9	454.6	372.4	− 18.1
34.2	204.2	343.0	68.0

## Conclusions and suggestions

Based on the principle of soil arching effect, the non-limit state earth pressure theory and horizontal differential element method are adopted to analyze the magnitude and distribution of the earth pressure on the sidewall of the open caisson, and the theoretical formula of the earth pressure on the sidewall of the open caisson is derived. The formula is suitable for working conditions where the sinking depth of the open caisson is large, the rate of mud absorption of the open caisson is small, or it is in a stagnant state. Based on the results of this article, selecting an appropriate sidewall friction coefficient of the caisson can calculate the frictional resistance value of the caisson sidewall, that providing an important basis for the design of the caisson and the stability judgment during the sinking process.

Through centrifugal model tests and theoretical calculations, it was found that when the embedded depth of the open caisson is large, the distribution of sidewall earth pressure of the open caisson first increases with the increase of embedded depth, reaches a peak value, and then sharply decreases. The peak point is located at 2/3 ~ 4/5 of the embedded depth.

The calculation model for the earth pressure on the sidewall of the open caisson established in this paper is simple and convenient with clear mechanical concept. In engineering practice, when the embedded depth of the open caisson is 40 m, the relative error between the field test value and the theoretical calculation value is − 55.8% ~ 1.2%, with an average error of 13.8%. When the equivalent embedded depth of the open caisson in the centrifugal model test is 36 m, the relative error between the centrifugal model test value and the theoretical calculation value is − 20.1% ~ 68.0%, with an average error of 10.6%.The calculation results are in good agreement with field measured results and the centrifugal model test results, which can provide reference for the design and construction of open caisson.

The distribution of earth pressure on the side wall of deep and large caisson is affected by the attitude of the caisson, and the change of the attitude of the caisson can be divided into two modes: rotation and translation. This paper only gives the theory of computation of the earth pressure distribution on the side wall when the caisson moves horizontally without tilting displacement during the sinking process. The theory of computation of the earth pressure distribution on the side wall when the caisson rotates and tilts is more complex, and needs further research.

## Data Availability

The data used to support the findings of this study are available from the corresponding author upon request.
